# Comparison of cNORM and LMS methods for estimating reference percentile curves from biometric data

**DOI:** 10.1038/s41598-025-29580-4

**Published:** 2025-11-27

**Authors:** Ronja Laurenz, Wolfgang Lenhard

**Affiliations:** 1 Institute of Psychology, Ruprecht-Karls-University, Heidelberg, Germany; 2 Institute of Psychology, Julius-Maximilians-University, Würzburg, Germany

**Keywords:** Data normalization, Reference percentile curves, Lambda Mu Sigma method, Taylor polynomial, Continuous norming, Biomarkers, Epidemiology, Software, Public health, Diagnosis

## Abstract

Accurate reference curves for biometric measures are essential for population health monitoring and screening. The *Lambda Mu Sigma Method (LMS)*, introduced by Cole and Green, is widely used in public health for generating age-specific reference percentiles. This study compares LMS with cNORM, a distribution-free approach based on Taylor polynomials, previously validated in psychometric applications. Using publicly accessible National Health and Nutrition Examination Survey (NHANES) datasets, we compared the performance of LMS and cNORM in modelling reference curves for body mass index (BMI) and maximum oxygen consumption (VO_2max_). We repeatedly drew random samples of different size to compute the models and cross-validated these to examine accuracy and bias of both methods across different percentile ranges. Performance metrics included *R*^*2*^, root mean square error and systematic deviation (Bias) from empirical percentiles. Both cNORM and LMS achieved high accuracy across the full distributions of BMI and VO₂_max_, but cNORM showed superior precision in extreme percentiles (± 2 SD), critical for identifying at-risk individuals. Accuracy improved with larger sample sizes, with a stronger effect for LMS, while interactions between method and sample size were dataset-specific and inconsistent. The distribution free approach implemented in cNORM offers a viable alternative to LMS for generating reference curves in public health applications, particularly when accurate classification in extreme ranges is crucial for screening decisions.

**Supplementary Information:**

The online version contains supplementary material available at 10.1038/s41598-025-29580-4.

## Approaches to reference percentile estimation

In the medical field, critical intervention decisions frequently rely on diagnostic assessments. When an absolute criterion is absent, normative reference values are employed to assess the severity of the results and their clinical implications. Specifically, the extreme expressions of biometric and medical features within a diagnostic context indicate the necessity for treatment. For example, diagnosing anorexia nervosa in adolescents involves identifying individuals whose body mass index (BMI) falls below the 5th percentile of sex- and age-standardized benchmarks, as outlined by the World Health Organization^1^. Conversely, obesity is determined by a BMI exceeding two standard deviations above the age- and sex-specific median^2^. Thus, establishing representative standardization samples is essential for accurately estimating age-specific distributions, but it is also essential to use suitable modelling techniques.

Statistical modelling of raw data distributions across ages, rather than solely depending on manifest data, offers several advantages, such as reducing sampling errors, enhancing the precision of reference norms, and facilitating the interpolation of reference percentiles for age groups not represented in the sample^3^. However, employing different statistical methodologies can produce varying results, leading to distinct reference percentiles based on the specific modelling techniques and parameters. This underlines the importance of meticulous selection and application of statistical approaches in generating clinically relevant reference values. Given the considerable impact of potential (mis)diagnoses, employing precise methodologies for estimating these standard values is crucial. The World Health Organization’s^4^ norm tables, for instance, were developed via the so-called LMS method. The name is derived from the parameters *Lambda*, *Mu* and *Sigma*, which are modelled by a Box–Cox power transformation^5,6^ to approximate a skewed normal distribution. In the last 15 years, this approach has profoundly advanced and extended with many different distribution families in the context of generalized additive models for location, scale and shape (GAMLSS;^7^). However, alternative methodologies exist that might exhibit advantageous characteristics for particular scenarios or sample sizes^8,9^.

### LMS (Lambda-Mu-Sigma) method

The LMS method^5,6^ represents a prevalent norming technique in percentile estimation. Rooted in the Box‒Cox transformation^10^ and enhanced by the application of the maximum penalized likelihood method, the LMS method has established its utility across a broad spectrum of research domains. It forms the basis of numerous diagnostic guidelines and is extensively applied in fields requiring the evaluation of anthropometric measures and medical indicators, such as blood pressure, fetal growth, malnutrition, and grip strength. The utility of the LMS method is particularly evident in situations where the variable of interest—be it body mass index, head circumference, or blood pressure—exhibits a strong dependency on an explanatory variable, typically age. It is critical to acknowledge the foundational assumptions underpinning the LMS method, as outlined by Cole^5^. First, the raw data utilized in the norming process are assumed to adhere to a skewed normal distribution, which can be rectified into a normal distribution through an appropriate power transformation. Thus, cases where the form of the raw score distribution fundamentally changes with age lead to a poor fit when LMS is used. Closely related to this is the communality assumption, positing that the spacing between individual percentile curves is proportional. Deviations from this assumption would manifest as adjacent percentile curves touching or overlapping, which would directly contribute to the theoretical framework^6^. Finally, the values to be modelled have to be strictly positive (> 0).

Derived from these principles, the distribution of raw scores corresponding to each specific value of the explanatory variable (*t*), such as for each age group, can be succinctly modelled using just three parameters. These parameters—*Lambda* (*L*), which represents the skewness of the distribution, *Mu* (*M,* representing the median) and *Sigma* (*S*, representing the spread)—provide the name of the LMS method. Theoretically, these parameters are uncorrelated and can be independently plotted against the explanatory variable, resulting in smooth trajectories over time. Together, they can approximate the raw value distribution within any given age segment^6^, and they can be modelled via polynomial regression or splines over age. The approach was methodologically advanced by Green, who proposed the application of the maximum-penalized likelihood estimation to jointly model *L*, *M*, and *S*. This method considers the entire dataset for estimation, thereby obviating the need for arbitrary age-based segmentation (Green in the discussion of Cole, 5).

#### Maximum penalized likelihood estimation

The “maximum likelihood” method aims to optimize parameter estimates and model intercepts to closely align the model with observed data, minimizing overfitting by favouring models that yield smooth percentile curve trajectories^11^. The *L(t)*, *M(t)*, and *S(t)* parameters are estimated by maximizing a *penalized likelihood* function, enhancing both the fit to the data and the smoothness of the *L*, *M*, and *S* curves(6, see Eq. (1)).

1 $$\begin{aligned} L\left( {L, M, S} \right) = & \mathop \sum \limits_{i = 1}^{n} \left( {L\left( {t_{i} } \right)log \left[ {\frac{{y_{i} }}{{M\left( {t_{i} } \right)}}} \right] - loglog S\left( {t_{i} } \right) - \frac{1}{2}\left\{ {\frac{{\left[ {\frac{{y_{i} }}{{M\left( {t_{i} } \right)}}} \right]^{{L\left( {t_{i} } \right)}} - 1}}{{L\left( {t_{i} } \right)S\left( {t_{i} } \right)}}} \right\}^{2} } \right) \\ & - \frac{1}{2}\alpha_{\lambda } \mathop \smallint \limits_{{}}^{{}} \left\{ {L^{\prime\prime}\left( t \right)} \right\}^{2} dt - \frac{1}{2}\alpha_{\mu } \mathop \smallint \limits_{{}}^{{}} \left\{ {M^{\prime\prime}\left( t \right)} \right\}^{2} dt - \frac{1}{2}\alpha_{\sigma } \mathop \smallint \limits_{{}}^{{}} \left\{ {S^{\prime\prime}\left( t \right)} \right\}^{2} dt \\ \end{aligned}$$

The penalized likelihood function includes three terms deducted from the likelihood, each involving the integral of the squared second derivative of a parameter function. The second derivative assesses the change in the slope of the parameter function at point *t*, serving as an indicator of the function’s unevenness. Consequently, the integral of the squared second derivative gauges the variation in the parameter function’s first derivative across the age range, with lower integral values indicating smoother parameter function curves^11^. These deductions from the likelihood effectively “penalize” rough parameter function estimates, allowing for adjustable smoothing levels through the selection of smoothing parameters by the researcher. A higher α results in smoother curves, which are represented as natural cubic splines with knots at each value of *t*^6^. Once estimates of the skewness, mean, and dispersion of the raw score distribution have been determined, a Box‒Cox transformation can be applied for each age stage to express the raw score distribution in adjusted SD scores, and these z scores can be used to determine the percentile curves.

#### Box‒Cox transformation

The Box‒Cox transformation facilitates the conversion of skew-normally distributed values into a standard normal distribution. By utilizing these known or estimated parameters for *Lambda*, *Mu* and *Sigma*, the transformation enables the calculation of standard deviation (SD) scores for raw scores across different values of the explanatory variable^5,10^; see Eqs. 2 and 3) and, consequently, percentiles via the cumulative density function of the normal distribution $F\left( z \right)$ (Eq. (4)).

2 $$z = \frac{{\left[ {y/M\left( t \right)} \right]^{L\left( t \right)} - 1}}{L\left( t \right)S\left( t \right)}, L\left( t \right) \ne 0$$

3 $$z = \frac{{log \left[ {y/M\left( t \right)} \right] }}{S\left( t \right)}, L\left( t \right) = 0$$

4 $$PR\left( z \right) = \frac{1}{{\sqrt {2\pi } }}\mathop \smallint \limits_{ - \infty }^{z} e^{{\frac{{ - t^{2} }}{2}}} dt$$

### cNORM—a distribution-free approach

Like the LMS method, cNORM models distributions across age groups simultaneously^6^, but differs in its distribution-free approach. The assumptions of LMS that raw values follow a skewed normal distribution transformable into a standard normal distribution can be violated in extreme trait expressions. This can lead to systematic errors, especially in the marginal ranges where discrepancies from the norm are most significant. These limitations become pronounced in psychometric scales prone to bottom or ceiling effects, where the LMS method’s efficacy diminishes, as demonstrated in simulation studies^12^. Parametric norming methods, including LMS, exhibit more substantial model deviations in extreme versus moderate trait expressions.

Several alternative methodologies have been proposed to address the limitations posed by violated distributional assumptions in raw value distributions. One is the non-parametric *Quantile Regression*^13^, which estimates age trajectories for each percentile independently through polynomial regression. This method does not rely on the parametric assumption about the distribution of raw values but requires large sample sizes and can lead to intersecting percentile curves. Another method implemented in *cNORM*^14,15^ utilizes higher-order Taylor polynomials to predict raw scores based on the explanatory variable (*a*) and location (Θ) of an individual within the reference group. This location parameter (Θ) represents an individual’s relative standing within their age-specific reference group. It is conceptualized as reflecting a normally distributed latent trait and can be expressed either as a percentile rank or as a standardized norm score derived from the normal rank transformation of that percentile. The explanatory variable (*a*) represents a systematically covarying factor related to the measured variable, such as age, in the case of variables such as body mass index (BMI) or cognitive ability, which typically increase monotonically throughout childhood and adolescence.

Distribution functions that are smooth throughout the measured range of values can be differentiated any number of times. Such functions can at any point be written as so-called Taylor series, which can approximate the original function extremely well over a sufficient range. This distribution-free approach combines modelling the distribution per reference group and the developmental trajectories over the explanatory variable simultaneously through two-dimensional Taylor polynomials, including powers of location $\Theta$ and explanatory variable $a$ (usually age) and all linear combinations. This resembles fitting a two-dimensional hyperplane (explanatory variable × location) in three-dimensional space^12^ to estimate the dependent raw scores (r). It does not rely on specific assumptions on the form of the distributions (Eq. (5)), with $c_{i, j}$ specifying the weight of the corresponding term in the regression function:

5 $$E(r|\Theta , a) = \mathop \sum \limits_{i = 0}^{k} \mathop \sum \limits_{j = 0}^{, t} c_{i,j} \Theta^{i} a^{j}$$

In cNORM, the power parameter *k* is set to 5 by default, which corresponds to the polynomial approximation of the location distribution, e.g., per age. The power parameter of the explanatory variable (e.g., age) *t* is set to 3 by default to capture curvilinear trajectories over age^15^. The *k* and *t* values act as smoothing parameters, with lower values leading to increased smoothing to balance the complexity of the model against the need to capture the underlying distributions accurately. In small datasets, it can be advisable to decrease these parameters to avoid overfitting. Increasing these values leads to a closer fit to the raw score distribution. In this way, $\left(k+1\right)\left(t+1\right)$ different terms are available for the modelling of the hyperplane. cNORM applies *best subset regression* to select the terms with the highest relevance^16,17^ and returns the best fitting model per number of terms in ascending order. cNORM per default returns the best fitting model, which passes initial checks of model coherence.

The established model predicts expected raw values as a function of an explanatory variable (e.g., age) and estimated person location. To convert raw scores at a specific age into norm scores, cNORM employs analytical methods to find the rational zero crossings of the first derivative of the regression function. If a unique solution is elusive, the algorithm resorts to a numerical approach, identifying values that most closely fit by minimizing the mean square error of the approximation^8^.

### Strengths and weaknesses of LMS and cNORM.

Continuous norming techniques, such as LMS and cNORM, address several crucial issues inherent in traditional reference table construction. The conventional approach to derive percentiles is based on the *Inverse Normal Transformation* (INT) of the empirical cumulative distributions. Thus, the discrete subsamples like age cohorts stand for themselves and all data, including sampling and measurement errors are used in the transformation. In contrast, continuous norming methods capture the development of the raw value distributions over explanatory variables like age and establish statistical models including the complete data. This leads to a considerable increase in statistical power^3^ and it offers several advantages: These methods eliminate gaps within and between norm tables, since norm scores can be calculated with the desired precision. They smooth out sampling errors in subsamples by drawing on the complete sample instead of isolated subsamples, prevent age-related biases because percentile can be retrieved for the exact age instead of broad age intervals. They reduce the risk of overfitting through manual control over of the degree of smoothing^3^. They require considerably smaller sample sizes compared to conventional percentile estimation, offering both, higher norm score quality and economic benefits^8^.

Parametric modelling, including the LMS method and similar distribution functions, is highly effective when the underlying assumptions are met. However, these assumptions may not hold if the measurement instruments, have a limited measurement range, leading to bottom and ceiling effects that alter the distribution type, making parametric modelling challenging. This is where distribution-free methods such as cNORM excel, as they lower the risk of model violations and systematic biases, particularly in the peripheral ranges critical for diagnosis^12^. In a simulation study comparing parametric and distribution-free approaches across varying sample sizes (n = 50—1000 per age group), cNORM produced more accurate models for smaller samples, whereas parametric models generally performed better with larger ones. Moreover, cNORM has recently been augmented with post stratification techniques to facilitate the establishment of representativeness in biased samples^18^.

As outlined above, the LMS method has been widely recognized and utilized across numerous studies, with Google Scholar reporting over 3000 citations for the seminal publication by Cole and Green^6^. cNORM has so far been employed predominantly in the development of various psychometric tests, including those for emotion regulation^19^, reading comprehension^20^, pronunciation^21^, vocabulary development^22^, motor skills^23,24^, dementia^25^, and general cognitive skills^26^, among others. A study on visual performance in macular degeneration^27^ has indicated that cNORM is capable of accurately estimating percentiles for medical and biometric data.

This study explores the application of the cNORM method to biometric data. While previous research has demonstrated cNORM’s utility in psychometric assessments, its potential for biometric norming has not yet been investigated. By systematically comparing cNORM to the well-established LMS approach, we aim to determine whether it provides more precise approximations of normed scores (e.g., age-specific percentiles, standard scores, etc.). Given its distribution-free nature, we hypothesize that cNORM outperforms the LMS method, particularly in regions of extreme feature expression (Hypothesis 1). Additionally, we assess its overall accuracy and bias across the full spectrum of biometric and medical data, investigating whether cNORM represents a superior alternative for norming in this domain (Hypothesis 2). On suggestion of the reviewers, we introduced a third, exploratory hypothesis regarding the size of the training data set: How does cNORM versus LMS perform using training data of differing size?

## Method

The use of freely available datasets with norm scores for biometric and medical measures previously estimated via the LMS method offers the possibility to address these hypotheses. We model these datasets via both LMS and cNORM for comparison. To ensure the robustness of our findings, we apply cross-validation to evaluate the predictive performance of each model. By calculating key metrics and conducting statistical analyses, we aim to identify any significant differences in the predictive abilities of LMS and cNORM.

### Samples

This study utilizes publicly accessible data from the National Health and Nutrition Examination Survey (NHANES), which was conducted in the United States. The NHANES 1999–2004 sample design utilized a multistage, stratified area probability approach to ensure a nationally representative sample of the U.S. noninstitutionalized civilian population^28^. Primary sampling units, mostly counties or groups of counties, were selected with probabilities proportional to the 2000 U.S. census. Within each unit, census blocks and newly constructed housing units were sampled, with oversampling of specific subpopulations, particularly Mexican–American individuals and other minority groups. In this study, NHANES serves as a reference dataset for comparing the performance of the modelling techniques rather than as a true population. Our aim is primarily to evaluate these methods in estimating percentiles within this sample independent of sampling considerations. We do not aim to establish population-representative reference values.

Laurson^29^ applied the LMS method to establish clinical BMI thresholds for diagnosing childhood obesity. This analysis incorporated data from the NHANES cycles of 1999–2000, 2001–2002, and 2003–2004, covering individuals aged 5;0–18;11 years^30^. After records with incomplete age and BMI data or those outside the specified age range were removed, the final sample comprised 9804 participants—4909 boys and 4895 girls – with an average age of *M* = 12.31 years and *SD* = 3.92 years (Sample 1).

Additionally, we considered maximal oxygen consumption (VO_2max_) by drawing upon the work of Eisenmann et al.^31^, who investigated this aspect in adolescents and utilized the LMS method to create percentile curves. The data for this analysis were sourced from the 1999–2000 and 2001–2002 NHANES cycles^30^, with a focus on the 12;0–18;11-year-old age group. Following the exclusion of incomplete datasets and those outside the target age range, the final cohort included 2997 subjects—1478 boys and 1519 girls – with an average age of *M* = 14.98 and *SD* = 1.98 (Sample 2). Notably, oxygen consumption is quantified in milliliters of oxygen per kilogram of body weight per minute. An overview of these datasets is provided in Table 1.

**Table 1:** Demographic data of the datasets.

Variable of interest	*N*_*female*_	*N*_*male*_	${M}_{age}$	${SD}_{age}$
Body-Mass-Index (Sample 1)	4895	4909	12.31	3.92
Maximum oxygen consumption (Sample 2)	1519	1478	14.98	1.98

Sample characteristics from NHANES datasets. N_female_ and N_male_ represent sample sizes for females and males respectively. M_age_ = mean age in years; SD_age_ = standard deviation of age in years.

### Study design

To compare how well both approaches approximate the empirical distributions of the test samples, we evaluated cNORM and LMS across different regions of the trait distribution. In addition to assessing overall predictive performance across the full range, we examined performance specifically within the extreme ranges of trait expression, where clinical decisions are often made. These extreme ranges encompass all measured values deviating by more than two standard deviations from the age-specific mean: values at or below − 2 SDs (lower extreme range) and values at or above + 2 SDs (upper extreme range).

The simulation was performed via the R platform, using the cNORM^14^ and gamlss^7^ packages in their current versions. For each simulation cycle, we computed the age-specific percentile and divided the samples into training and testing datasets via repeated stratified random sampling: We drew 50, 100, 150 or 200 cases per age cohort (spanning one year each) as the training data and used the remaining cases as the test data. In case of the VO_2max_ data, which included 7 age cohorts, the training set amounted to 350, 700, 1050 and 1400 cases, representing a share of 11.6%, 23.3%, 35.0% or 46.7% of the data (the rest serves as test data). In case of the BMI data with their 14 age cohorts, this amounted to 700, 1400, 2100 or 2800 cases, representing 6.9%, 13.8%, 20.7% or 27.8% of the data – again with the rest serving as test data. We computed statistical models via the *lms* function (gamlss package) or *cnorm* (cNORM package) in their default configuration based on the training data and predicted the percentiles for the test data. (code provided in the OSF repository). The *lms* function models the data via the original Cole and Green^6^ approach (function family ‘BCCGo’). *cnorm* applies a model selection approach based on *best subset regression* of the Taylor polynomials.

We repeated the process for each of the four factor levels (50, 100, 150 or 200) of the training sample size 1,000 times via random sampling to evaluate the predictive accuracy of the different models. This resulted in 4,000 runs each for the BMI and the VO_2max_ samples. The norm scores are expressed as *T* scores (*M* = 50, *SD* = 10). To compare the two methods’ predictive ability, we calculated *R*^*2*^, the root mean square error (*RMSE*, Eq. 6) and *Bias* (Eq. 7). While the *RMSE* provides a measure of how well a model can predict the outcome variable, *Bias* indicates systematic divergences of the predicted data from the actual data. Precision measures were obtained by deriving *T*_*actual*_ via an inverse normal-rank transformation (INT) of the empirical cumulative distribution function (eCDF) of the complete data, which served as the benchmark for *T*_*predicted*_ estimated by the cNORM and LMS approaches (*n* denotes the sample size).

6 $$RMSE = \sqrt {\frac{1}{n}\mathop \sum \limits_{i = 1}^{n} \left( {T_{actual} - T_{predicted} } \right)^{2} }$$

7 $$Bias = \frac{1}{n}\mathop \sum \limits_{i = 1}^{n} \left( {T_{actual} - T_{predicted} } \right)$$

These indicators for the predictive power of the models were not only calculated for the complete datasets but also for the age-specific extreme manifestations.

### Statistical analysis

**Fig. 1:**
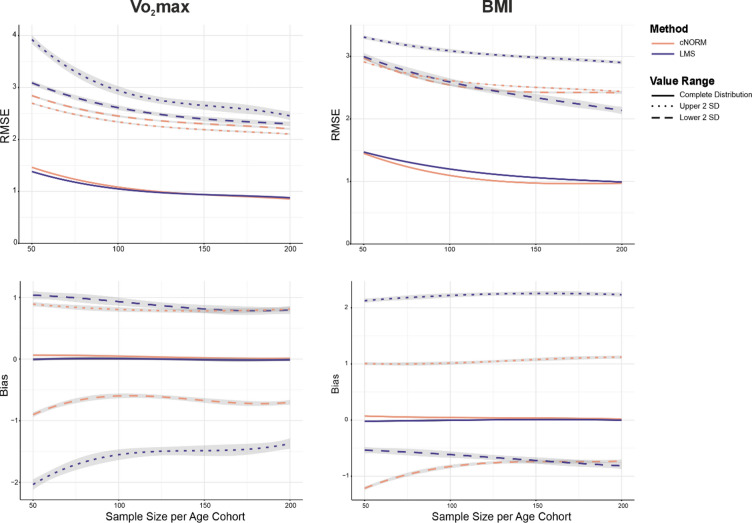
*RMSE* and *Bias* in modelling the BMI and the VO_2max_ data. *Notes*. The figure depicts the results of modelling BMI and VO_2max_ data with cNORM (orange) versus LMS (blue). The solid lines represent the results for the complete sample, the dotted lines the lower tails of the distributions (− 2 *SD* and lower) and the dashed lines the upper tail of the distributions (+ 2 *SD* and higher). The grey intervals indicate the confidence intervals including ± 1 1 *SE*. The results are reported as *T* scores (*M* = 50, *SD* = 10). Due to the standardization, the absolute values and the differences between the methods can be interpreted as effect sizes sensu *d*_*Cohen*_ by dividing them by 10.

To test our hypotheses, we employed linear mixed-effects models (LMMs). These models examined the main effects of method and sample size, as well as the interaction effect between method and sample size. Separate models were fitted for each dataset and each outcome variable, namely *RMSE*, *Bias*^2^ and *R*^2^. To avoid a high number of tests for different directions, we squared the *Bias*. The unsquared results are shown in Fig. 1. For *RMSE* and *Bias*^2^, additional models were estimated separately for the lower, upper, and complete measurement domains. To evaluate the overall performance of cNORM compared with LMS, we examined the main effects of the method in our models for each criterion within the complete measurement range. For all analysis, the condition with Method = cNORM and Sample = 50 was defined as the baseline category. We report effect sizes for direct comparisons between cNORM and LMS. Moreover, since the *RMSE* values were computed from standardized scores, they also serve as direct measures of effect size.

Following the 4,000 repetitions of the random sampling, a large sample is available. To estimate the lower bounds of the statistical power, we computed 1—*β* using G*Power for main effects in regression analyses. With an *α* = 0.001 we can identify small effects of d = 0.3 with a power of 1—*β* > 0.999. Since linear mixed-effects models have an even superior power, we restricted our interpretation to highly significant effects.

## Results

### Effects of modelling approach

In evaluating the overall efficacy of modelling BMI and VO_2max_ data, cross-validated predictions of norm scores in the test datasets revealed small *RMSE* values for both methods (Electronic Support Material ESM1 Table S1 and S2; Fig. 1). Given that all values reported here are scaled as *T* scores, these can easily be interpreted as effect sizes, and the same is true for their differences. Dividing them by a factor of 10 yields a *d*_*Cohen*_ comparable metric.

The analyses of the method effects of *RMSE*, *Bias*^*2*^, and *R*^2^ values across the full data spectrum showed small mixed effects and null effects across both datasets, with only minor *RMSE* and *Bias*^*2*^ values and high *R*^2^ values for both methods. In the VO_2max_ dataset, the *LMS* method achieved slightly lower *RMSE* and higher *R*^2^ values, *RMSE t*(6 997) = -10.606, *p* < 0.001; *R*^2^ *t*(6 997) = 20.29, *p* < 0.001. In contrast, in the BMI dataset, *cNORM* achieved slightly lower *RMSE* and higher *R*^2^ values, *RMSE t*(6 997) = 7.350, *p* < 0.001; *R*^2^ *t*(6 997) = − 7.574, *p* < 0.001. The method effects for *Bias*^*2*^ were not significant for either dataset and the effect sizes were generally very small.

Regarding the lower and upper measurement level, we found substantial differences for both methods in both datasets, indicating that *LMS* produced significantly higher *RMSE* and *Bias*^*2*^ values, especially for the VO_2max_ data: *RMSE*_*lower*_* t*(6 997) = 8.834, *p* < 0.001; *Bias*^*2*^_*lower*_* t*(7 996) = 17.364, *p* < 0.001; *RMSE*_*upper*_* t*(6 997) = 25.41, *p* < 0.001 and *Bias*^*2*^_*upper*_* t*(7 996) = 25.187, *p* < 0.001. In the BMI dataset, the method effects followed a similar pattern, *RMSE*_*lower*_* t*(6 997) = 4.889, *p* < 0.001; *Bias*^*2*^_*lower*_* t*(6 997) = -0.383, *p* = 0.702; *RMSE*_*upper*_* t*(6 997) = 19.77, *p* < 0.001 and *Bias*^*2*^_*upper*_* t(*6 997) = 35.109, *p* < 0.001. All highly significant method effects were positive, indicating that LMS norming produced higher *RMSE* and *Bias*^*2*^ values. Only the method effect for *Bias*^*2*^_*lower*_ in the BMI data was negative and not significant. Except for this non-significant effect, all remaining highly significant method effects in both the lower and upper measurement levels support the hypothesis that cNORM provides more accurate modelling in extreme feature ranges compared to LMS.

### Effects of sample size

 We found a highly significant main effect of sample size: Increasing the sample size leads to more accurate estimation of normative values across all feature domains and for both norming methods. With larger sample size, *RMSE* and *Bias*^*2*^ decrease, while *R*^2^ increases (ESM1 Table S1 and S2, Fig. 1). Only in case of the main effect estimates of sample size for *Bias*^*2*^_*upper*_ in the VO_2max_ dataset, and for *Bias*^*2*^_*upper*_ in the BMI dataset, this was not true. For all remaining main effects of sample size, the pattern is consistent.

Mixed patterns and dataset-specific differences emerged when examining the interaction between sample size and method. Unlike the VO_2max_ dataset, interactions in the BMI data were generally weak or inconsistent. Several highly significant interactions indicate that LMS shows a stronger response to increasing sample size than cNORM, especially in the extreme ranges of the VO_2max_ dataset, *RMSE*_*lower*_* t*(6 997) = − 4.474, *p* < 0.001; *Bias*^*2*^_*lower*_* t*(7 996) = − 10.328, *p* < 0.001; *RMSE*_*upper*_* t*(6 997) = − 14.23, *p* < 0.001; *Bias*^*2*^_*upper*_* t*(7 996) = − 14.428, *p* < 0.001, but as well for the lower ranges of the BMI data, *RMSE*_*lower*_* t*(6 997) = − 7.490, *p* < 0.001. In contrast, cNORM benefitted more regarding *RMSE* in the VO_2max_ dataset, *t*(6 997) = 9.238, *p* < 0.001, and the lower part of the BMI dataset, *Bias*^*2*^_*lower*_* t*(7 996) = − 7.932, *p* < 0.001.

To sum up, both models show a good performance already with small sample sizes, but profit from larger samples. Increasing the sample size leads to overall higher precision with a stronger effect on LMS. The interaction patterns are however complex, inconsistent and dependent on the dataset.

## Discussion

The objective of this study was to evaluate the suitability of the continuous norming method cNORM for percentile modelling in biometric data, specifically through empirical datasets on body mass index (BMI) and oxygen consumption (VO_2max_), and to contrast its efficacy with the established LMS method. Our focus was to assess the predictive performance across the entire data spectrum and within extreme feature expressions, employing random sampling and cross-validation. We further examined how the sample size of the training data affects the accuracy of normative estimates for both methods.

Across the full range of feature expression, both methods demonstrated competent modelling capabilities, characterized by minimal *RMSE*s in both datasets, high *R*^2^ values and negligible *Bias*. While the VO_2max_ dataset showed a slight advantage for LMS, cNORM exhibited greater overall accuracy in modelling BMI data. Consequently, these mixed results do not support our hypothesis, which assumed superior performance of cNORM across all feature domains. Both methods are equally suited for the complete feature range. In extreme feature ranges, cNORM overall showed lower *RMSE* and *Bias*^*2*^ than LMS across both datasets, supporting the hypothesis on the improved accuracy of cNORM in modelling the distribution tails. Despite its psychometric origins, cNORM can be effectively applied to biometric data, underscoring its versatility and precision across diverse analytical contexts.

Considering the influence of training sample size, the methods profited from an increasing sample size across both datasets. Examination of the interaction effects shows that most interpretable interactions suggest LMS benefits more from larger sample sizes, particularly in estimating norms for the extreme feature ranges of the VO_2max_ dataset, which is due to poorer performance of this method in small samples (*n* = 50 per age cohort). However, no consistent interaction effect favours one method over the other. Measurement precision depends on the use case; thus no definitive cutoff can be set. But, since the largest improvement in model precision occurred between *n* = 50 and *n* = 100, age cohorts of 100 can probably be seen as both necessary and sufficient for percentile estimation.

To frame our results within the broader research context, we revisit the study’s hypotheses and their theoretical underpinnings. A key advantage of the cNORM methodology over the LMS method is its distribution-independent nature, which does not presuppose a specific distribution for raw values^12^. This characteristic is particularly beneficial because the distribution assumptions inherent to LMS may not hold as well across all trait’s extreme ranges^15^. Consequently, we anticipated that cNORM would demonstrate superior performance to parametric methods in modelling extreme feature expressions, which are often of critical diagnostic significance. Since, in these extreme feature expressions, considerably fewer cases are available, modelling is inherently affected by a decreased accuracy and consequently larger *RMSE* values. As expected, this is true for all the methods presented in this paper. Notably, cNORM was less affected by this imprecision than LMS.

Moreover, the ability of cNORM to model with high statistical power is attributed to its simultaneous horizontal and vertical interpolation of the hyperplane. This approach enables precise predictions for the feature of interest within the population, even with limited sample sizes^3,8^. Based on this theoretical framework, our second hypothesis posited that cNORM would outperform LMS in overall model predictions across all feature domains. However, the findings of this study did not fully support this hypothesis. Instead, we observed that cNORM and LMS, particularly when the gamlss package was used for LMS-based modelling, produced nearly identical predictions within moderate measurement ranges. In the VO_₂max_ dataset, LMS achieved slightly more accurate norm value estimations, whereas in the BMI dataset, cNORM showed a modest advantage. Despite observable differences in norming errors across the datasets, the disparity in model predictive performance is minimal. Consequently, for predictions spanning the entire feature range, both methods demonstrate equivalent performance in modelling the biometric datasets. Although this outcome challenges the study’s second hypothesis, it does not diminish the applicability of cNORM for normalizing biometric data, as model predictions across the full feature spectrum are comparably effective.

### Limitations

Notably, the LMS method has been further developed by applying maximum-penalized likelihood estimation, which elegantly solves some of the issues with the original LMS method and incorporates the dataset as a whole in the modelling^6^. Nevertheless, there is the possibility that some raw distribution assumptions are violated, which makes precise modelling challenging in the impacted regions. The differences between the two norming approaches could have been greater if the original LMS method had been used for the method comparison in this study.

It is necessary to critically examine a few of the investigation’s methodological choices. For example, *i* = 1000 was arbitrarily chosen as the number of random sampling repetitions, yielding a total of 4000 observations for the *Bias* and *RMSE* for each dataset. Owing to the large number of observations, statistical power increases significantly when these features are analysed statistically and can be enhanced arbitrarily with any choice of *i*. This makes even seemingly trivial effects potentially significant. The issue of sometimes questionable practical relevance of the significant differences found must be interpreted based on effect size, which is one consequence of very high test power.

Another issue is that we did not fine-tune the automatically generated models, so they were compared with each other via default settings. This further weakened method effects, hindered the full potential of the standardizations, and resulted in the inclusion of less-than-ideal models in the comparison. In real scenarios, researchers will most probably fine tune the model by hand, delete implausible models and find superior solutions to the automatically retrieved models.

An additional critique is that the test dataset’s manifest T score is taken to represent the individual’s latent feature expression, which was determined by transforming the raw scores via an inverse normal-rank transformation. While in psychometrics, a normally distributed latent feature expression is assumed, this premise does not necessarily hold for biometric data. Given the modelling results, this premise is obviously not necessary for data modelling purposes.

Finally, it should be noted that the choice of biometric datasets was largely influenced by the degree to which researchers who used the LMS method to normalise biometric data made their raw data accessible. Future research could perform comparative work using different publicly available datasets, avoiding the limitation of only observing data that have already been normalised with LMS.

### Conclusion

The comparable modelling performance of cNORM in comparison to LMS over the whole dataset and the superior precision in extreme feature expressions make cNORM a suitable method to data modelling in the biometric field. As both methods benefit from larger sample sizes, the differences diminish when larger normative datasets are employed. From the standpoint of practical considerations, using datasets of 100 cases or more per age cohort generally lead to precise statistical models with both methods. The choice of sample size however depends on the trajectory of the development of the feature over age and it is advisable to provide better coverage for age intervals with rapid changes.

Raising diagnostic standards is highly relevant from a practical standpoint since most medical decisions in the healthcare industry are based on the interpretation of test and measurement results via norm tables. Accurate norm tables at the clinically relevant extents of a characteristic enable a confident diagnosis regarding the need for medical intervention. As norming methods influence medical decisions, selecting and applying the best suitable modelling technique for specific use cases is crucial. cNORM might enrich the statistical method inventory and help cases that are hard to model parametrically.

## Supplementary Information

Below is the link to the electronic supplementary material.

Supplementary Material 1

## Data Availability

All the data and syntax files are available from the OSF database via https://osf.io/xtnkd
