# Supplementary material for: Comparison of cNORM and LMS methods for estimating reference percentile curves from biometric data
Source: Sci Rep. 2025 Nov 27;15:42561. doi: 10.1038/s41598-025-29580-4 (PMC12663128; doi:10.1038/s41598-025-29580-4)
Supplement: Supplementary file 1 — Supplementary Material 1 [file 41598_2025_29580_MOESM1_ESM.docx]

**Table S1. Model estimates for VO2max fit indices (RMSE, Bias², R²) by method and sample.**

|  |  | VO2max | | | | | |
| --- | --- | --- | --- | --- | --- | --- | --- |
|  |  | | *Estimates* | *CI* | *df* | *t* | *p* |
| *RMSE* |  |  | |  |  |  |  |
| complete | (Intercept) | 1.579e+00 | | 1.56 – 1.59 | 7 990 | 208.026 | **<0.001** |
|  | Method [LMS] | -1.106e-01 | | -0.13 – -0.09 | 6 997 | -10.606 | **<0.001** |
|  | Sample | -3.951e-03 | | -0.00 – -0.00 | 6 997 | -73.338 | **<0.001** |
|  | Method[LMS]:Sample | 7.037e-04 | |  | 6 997 | 9.238 | **<0.001** |
| lower | (Intercept) | 2.968e+00 | | 2.92 – 3.01 | 7 959 | 132.156 | **<0.001** |
|  | Method [LMS] | 2.777e-01 | | 0.22 – 0.34 | 6 997 | 8.834 | **<0.001** |
|  | Sample | -4.145e-03 | | -0.00 – -0.00 | 6 997 | -25.541 | **<0.001** |
|  | Method[LMS]:Sample | -1.027e-03 | | -0.00 – -0.00 | 6 997 | -4.474 | **<0.001** |
| upper | (Intercept) | 2.815e+00 | | 2.74 – 2.89 | 7 949 | 74.10 | **<0.001** |
|  | Method [LMS] | 1.353e+00 | | 1.25 – 1.46 | 6 997 | 25.41 | **<0.001** |
|  | Sample | -3.861e-03 | | -0.00 – -0.00 | 6 997 | -14.04 | **<0.001** |
|  | Method[LMS]:Sample | -5.533e-03 | | -0.01 – -0.00 | 6 997 | -14.23 | **<0.001** |
| *Bias²* |  |  | |  |  |  |  |
| complete | (Intercept) | 2.912e-01 | | 0.28 – 0.31 | 7 854 | 36.732 | **<0.001** |
|  | Method [LMS] | 9.429e-03 | | -0.01 – 0.03 | 6 997 | 0.883 | 0.377 |
|  | Sample | -1.410e-03 | | -0.00 – -0.00 | 6 997 | -25.577 | **<0.001** |
|  | Method[LMS]:Sample | -4.204e-05 | | -0.00 – 0.00 | 6 997 | -0.539 | 0.590 |
| lower | (Intercept) | 1.586e+00 | | 1.41 – 1.77 | 7 996 | 17.215 | **<0.001** |
|  | Method [LMS] | 2.262e+00 | | 2.01 – 2.52 | 7 996 | 17.364 | **<0.001** |
|  | Sample | -5.337e-03 | | -0.01 – -0.00 | 7 996 | -7.932 | **<0.001** |
|  | Method[LMS]:Sample | -9.827e-03 | | -0.01 – -0.01 | 7 996 | -10.328 | **<0.001** |
| upper | (Intercept) | 1.638e+00 | | 1.23 – 2.05 | 7 996 | 7.858 | **<0.001** |
|  | Method [LMS] | 7.423e+00 | | 6.84 – 8.00 | 7 996 | 25.187 | **<0.001** |
|  | Sample | -4.832e-03 | | -0.01 – -0.00 | 7 996 | -3.175 | 0.0015 |
|  | Method[LMS]:Sample | -3.105e-02 | | -0.04 – -0.03 | 7 996 | -14.428 | **<0.001** |
| *R²* |  |  | |  |  |  |  |
| complete | (Intercept) | 9.802e-01 | | 0.98 – 0.98 | 7 996 | 6610.73 | **<0.001** |
|  | Method [LMS] | 4.158e-03 | | 0.00 – 0.00 | 6 997 | 20.29 | **<0.001** |
|  | Sample | 7.162e-05 | | 0.00 – 0.00 | 6 997 | 67.68 | **<0.001** |
|  | Method[LMS]:Sample | -2.356e-05 | | -0.00 – -0.00 | 6 997 | -15.74 | **<0.001** |

*Note*. Analyses are based on *n* = 4000, corresponding to 8000 observations in long format for the linear mixed-effects model analyses. Shown are linear mixed-effects model estimates for the main effects of method and sample size, as well as their interaction, across model fit indices (RMSE, Bias², R²) for the complete data and for the lower (−2 SD) and upper (+2 SD) feature ranges. Reported values include estimates, 95% confidence intervals (*CI*), degrees of freedom (*df*), *t*- and *p*-values. The estimates for RMSE and Bias are based on standardised scores with *SD* = 10. Thus, dividing them by 10 can be interpreted as effect sizes.

**Table S2. Model estimates for BMI fit indices (RMSE, Bias², R²) by method and sample.**

|  |  | BMI | | | | | |
| --- | --- | --- | --- | --- | --- | --- | --- |
|  |  | | *Estimates* | *CI* | *df* | *t* | *p* |
| *RMSE* |  |  | |  |  |  |  |
| complete | (Intercept) | 1.512e+00 | | 1.50 – 1.52 | 7 930 | 249.863 | **<0.001** |
|  | Method [LMS] | 6.249e-02 | | 0.05 – 0.08 | 6 997 | 7.350 | **<0.001** |
|  | Sample | -3.114e-03 | | -0.00 – -0.00 | 6 997 | -70.924 | **<0.001** |
|  | Method[LMS]:Sample | -3.556e-05 | | -0.00 – 0.00 | 6 997 | -0.573 | 0.567 |
| lower | (Intercept) | 3.037e+00 | | 2.98 – 3.09 | 7 892 | 111.581 | **<0.001** |
|  | Method [LMS] | 1.878e-01 | | 0.11 – 0.26 | 6 997 | 4.889 | **<0.001** |
|  | Sample | -3.570e-03 | | -0.00 – -0.00 | 6 997 | -18.001 | **<0.001** |
|  | Method[LMS]:Sample | -2.101e-03 | | -0.00 – -0.00 | 6 997 | -7.490 | **<0.001** |
| upper | (Intercept) | 3.003e+00 | | 2.97 – 3.03 | 7 934 | 209.71 | **<0.001** |
|  | Method [LMS] | 3.977e-01 | | 0.36 – 0.44 | 6 997 | 19.77 | **<0.001** |
|  | Sample | -3.084e-03 | | -0.00 – -0.00 | 6 997 | -29.70 | **<0.001** |
|  | Method[LMS]:Sample | 4.421e-04 | | 0.00 – 0.00 | 6 997 | 3.01 | 0.0026 |
| *Bias²* |  |  | |  |  |  |  |
| complete | (Intercept) | 1.745e-01 | | 0.17 – 0.18 | 7 884 | 38.031 | **<0.001** |
|  | Method [LMS] | -5.781e-03 | | -0.02 – 0.01 | 6 997 | -0.933 | 0.351 |
|  | Sample | -8.139e-04 | | -0.00 – -0.00 | 6 997 | -25.441 | **<0.001** |
|  | Method[LMS]:Sample | 3.594e-05 | | -0.00 – 0.00 | 6 997 | 0.794 | 0.427 |
| lower | (Intercept) | 2.118e+00 | | 2.01 – 2.23 | 7 942 | 36.994 | **<0.001** |
|  | Method [LMS] | -3.075e-02 | | -0.19 – 0.13 | 6 997 | -0.383 | 0.7018 |
|  | Sample | -8.362e-03 | | -0.01 – -0.01 | 6 997 | -20.159 | **<0.001** |
|  | Method[LMS]:Sample | 2.216e-03 | | 0.00 – 0.00 | 6 997 | 3.778 | **<0.001** |
| upper | (Intercept) | 1.398e+00 | | 1.24 – 1.56 | 7 985 | 16.964 | **<0.001** |
|  | Method [LMS] | 4.026e+00 | | 3.80 – 4.25 | 6 997 | 35.109 | **<0.001** |
|  | Sample | -4.566e-04 | | -0.00 – 0.00 | 6 997 | -0.771 | 0.441 |
|  | Method[LMS]:Sample | -7.267e-04 | | -0.00 – 0.00 | 6 997 | -0.868 | 0.386 |
| *R²* |  |  | |  |  |  |  |
| complete | (Intercept) | 9.801e-01 | | 0.98 – 0.98 | 7 891 | 6929.572 | **<0.001** |
|  | Method [LMS] | -1.512e-03 | | -0.00 – -0.00 | 6 997 | -7.574 | **<0.001** |
|  | Sample | 6.318e-05 | | 0.00 – 0.00 | 6 997 | 61.282 | **<0.001** |
|  | Method[LMS]:Sample | 2.297e-06 | | -0.00 – 0.00 | 6 997 | 1.576 | 0.115 |

*Note*. Analyses are based on *n* = 4000, corresponding to 8000 observations in long format for the linear mixed-effects model analyses. Shown are linear mixed-effects model estimates for the main effects of method and sample size, as well as their interaction, across model fit indices (RMSE, Bias², R²) for the complete data and for the lower (−2 SD) and upper (+2 SD) feature ranges. Reported values include estimates, 95% confidence intervals (*CI*), degrees of freedom (*df*), *t*- and *p*-values. The estimates for RMSE and Bias are based on standardised scores with *SD* = 10. Thus, dividing them by 10 can be interpreted as effect sizes.
